# Effects of fertilization measures on soil fertility and crop yields in drylands of the North China Plain: a meta-analysis

**DOI:** 10.3389/fpls.2026.1768991

**Published:** 2026-02-11

**Authors:** Yi Chang, Xianmei Zhang, Qing Shu, Zihao Sun, Mei Yang, XiaoYan Liu, Jinpeng Guo, Zhenquan He, Ling Li, Jing Huang, Jinhu Zhi, Guodong Chen, Tiantao Wang

**Affiliations:** 1 College of Agriculture, Tarim University, Alar, China; 2 State Key Laboratory of Efficient Utilization of Arable Land in China, Key Laboratory of Arable Land Quality Monitoring and Evaluation, Ministry of Agriculture and Rural Affairs, The Institute of Agricultural Resources and Regional Planning, Chinese Academy of Agricultural Sciences, Beijing, China; 3 College of Biology and Resource Environment, Beijing Agricultural College, Beijing, China; 4 School of Biological Sciences and Agriculture, Honghe University, Mengzi, China; 5 College of Horticulture, Ludong University, Yantai, China; 6 College of Resources and Environment, Shandong Agricultural University, Taian, China; 7 R&D Department, Zibo Qingda Powder Materials Engineering Co., Ltd., Zibo, China

**Keywords:** crop yield, fertilization practices, meta-analysis, North China Plain, soil fertility

## Abstract

This study aims to quantify the effects of three fertilization patterns—“chemical fertilizer alone,” “organic fertilizer alone,” and “mixed application of organic and inorganic fertilizers”—on soil fertility and crop yields. It also assesses the relationships among baseline soil properties, climatic factors, and fertilization effects in the North China Plain. By compiling relevant literature on these three fertilization patterns over the past 30 years in the region and employing meta-analysis methods, this study incorporated 63 wheat-related studies (1,065 datasets) and 34 maize-related studies (631 datasets). The analysis examined the effects of different fertilization methods on crop yield, soil nutrients, microbial carbon and nitrogen content, and soil enzyme activity, as well as the relationships between baseline soil properties, climatic factors, and fertilization outcomes. Results revealed: 1) Short-term application of chemical fertilizers and organic-inorganic composite fertilizers significantly increased wheat yields more than maize yields, whereas organic fertilizer application boosted maize yields more than wheat yields. Organic-inorganic composite fertilization most significantly enhanced soil microbial biomass and key soil enzyme activities, while chemical fertilizer alone showed the weakest improvement effects. 2) Fertilization impacts yield under baseline conditions of soil pH 6–8, soil organic matter content below 15 g/kg, annual mean temperature of 13°C–14°C, and annual precipitation exceeding 500 mm. 3) Long-term application of chemical fertilizers alone shows slowing yield increases, whereas organic-inorganic composite fertilization sustainably boosts yields and mitigates the impact of single factors on production. Unified comprehensive fertilization rates for wheat and maize are recommended as follows: nitrogen fertilizer application of 150–225 kg·ha^⁻¹^, phosphorus fertilizer of 60–90 kg·ha⁻¹, potassium fertilizer of 30–60 kg·ha⁻¹, supplemented with 7.5–9 tons·ha⁻¹ of organic fertilizer. This approach outperforms single-fertilizer regimes in improving soil microbial properties and enzyme activity, serving as the key to superior yield maintenance. The recommended integrated fertilization rates strike a balance between reducing chemical fertilizer use, stabilizing yields, and enhancing soil fertility, providing practical guidance for fertilizer management in the North China Plain.

## 1 Introduction

Food supply security is a crucial foundation for a nation’s long-term peace and stability. As China’s primary wheat-producing region, the North China Plain accounts for approximately 80% of the country’s wheat supply and contributes 14% to global wheat production (Liu et al., 2025). This plain ranks among the world’s most densely populated and intensively farmed areas, encompassing 25% of China’s arable land. The dominant cropping pattern in the region is a winter wheat–summer maize double-cropping system (Zhang et al., 2022). Fertilizer application is a widely adopted practice for maintaining soil fertility (Guan et al., 2023). Since the 1980s, the fields of the North China Plain have relied on an intensive “high-input, high-output” fertilization model, with fertilizer application driving annual crop yield increases. Data from the National Bureau of Statistics of China indicate that fertilizer application intensity in this region reaches 350–450 kg·ha^−1^, far exceeding the national average. However, crop yields have not increased in tandem with fertilizer application levels (Wang et al., 2009). The yield-enhancing effect of fertilization and marginal value-added output are both showing a declining trend (Zhang et al., 2020) while also leading to soil acidification and salinization (Wambacq et al., 2022).

Soil fertility is the fundamental basis of agricultural production and is primarily determined by the effective supply of readily available carbon (C) and nitrogen (N) in soil. The balance between these two elements directly influences the immediate availability of nutrients to crops (Barlog, 2023). Soil organic C (SOC) is a key regulator of soil fertility, sustaining soil ecosystem services and enhancing crop growth and yield by improving soil structure and regulating the chemical environment (Liu et al., 2025). Additionally, the ratio of soil organic carbon to total nitrogen (TN) serves as a key quantitative indicator for assessing soil fertility. These parameters reflect both soil nutrient reserves and supply potential, serving as essential metrics for evaluating farmland ecosystem stability and providing reliable support for agricultural production (Wang et al., 2022). Microbial C and N constitute the core components of the active soil C and N pools, converting complex organic C and N into readily available nutrients for plants. Soil enzyme activity regulates the transformation of organic nutrients into accessible forms, reflecting microbial community function, maintaining community balance, and supporting stable soil ecological processes (Xing et al., 2025).

Current fertilization practices in the North China Plain exhibit considerable diversity. Existing studies reveal significant variability in the conclusions regarding the effects of different fertilization methods. Moreover, individual field trials are limited by regional factors, climatic conditions, and cropping history, making it challenging to identify universal patterns across various fertilization practices. This study used a meta-analysis to quantitatively assess the effects of fertilization practices on the yields of wheat and maize, which are the two major grain crops in the North China Plain. It examined the effects of different fertilization approaches on agricultural productivity, nutrient accumulation, and sustainability. The aim of this study was to provide scientific evidence to optimize fertilization strategies in the North China Plain, support the implementation of fertilizer reduction policies, and promote sustainable agricultural development in the region.

## 2 Materials and methods

### 2.1 Data collection

**Figure 1:**
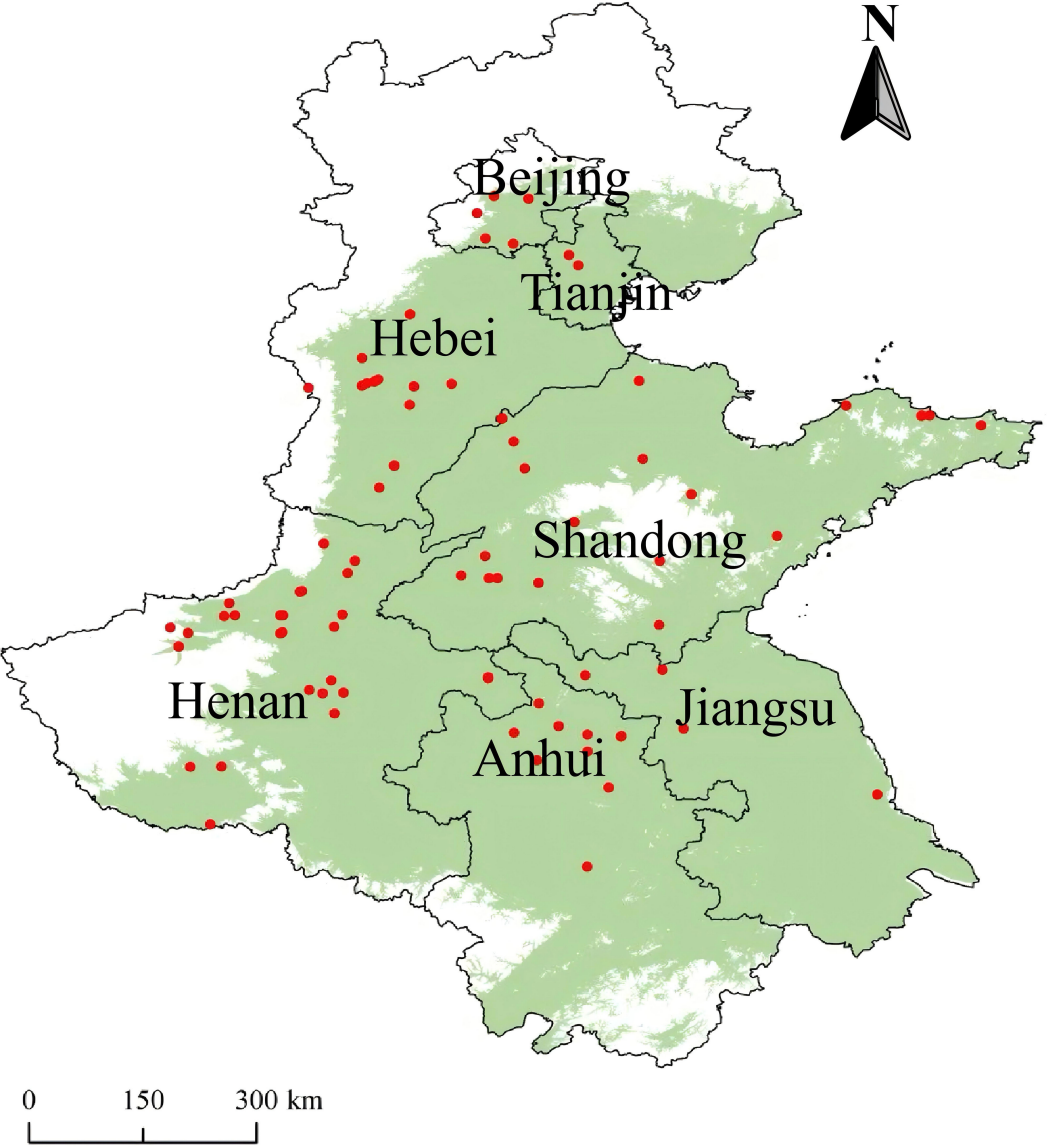
Location of the studies included in the meta-analysis.

**Figure 2:**
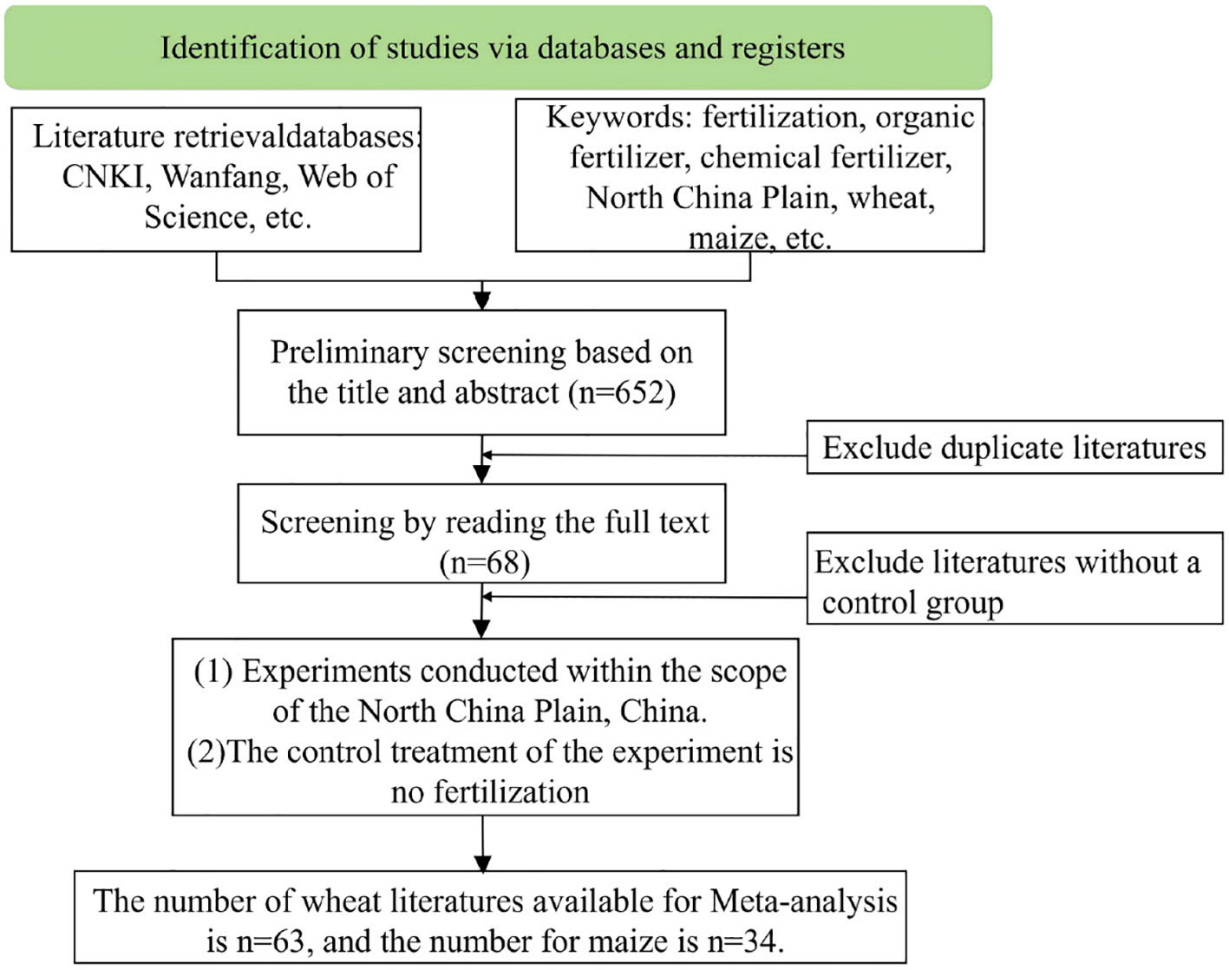
Literature screening process.

Literature searches were conducted using the Chinese National Knowledge Infrastructure, Wanfang, and the Web of Science. The selected keywords include “fertilization,” “chemical fertilizers (CF),” “organic fertilizers (OF),” “combined application of organic and inorganic fertilizers (COF),” “North China Plain,” “wheat and maize,” and “yield,” with a time span from 1995 to 2025. Based on the data integration requirements for meta-analysis methods and the objectives of this study, the literature was screened according to the following criteria: 1) field trials conducted in provinces and cities within the North China Plain, reporting basic information, such as start and end years, location, latitude and longitude, and physicochemical properties prior to the experiment; 2) crops included at least wheat, maize, or both; 3) each study contained both unfertilized and fertilized treatments (organic fertilizer, chemical fertilizer, or combined organic–inorganic fertilizer), with consistent non-fertilizer field management practices across treatments; 4) mean yield and standard deviation were either directly obtainable or calculated; and 5) data were extracted using PlotDigitizer, including details on replication numbers, mean values, and measures of variability [standard error (SE) or standard deviation (SD)]. If the standard deviation is not provided in the literature, it is assumed to be 1/10 of the mean (Tian et al., 2015). Applying these criteria yielded 63 wheat studies with 1,065 datasets and 34 maize studies with 631 datasets covering the North China Plain region (**Figure 1**). The screening process is illustrated in **Figure 2**.

This study also collected soil and climate data, along with other experimental design parameters that influenced the treatment effect values. These parameters included experimental location (latitude and longitude), soil properties (soil texture, pH, organic C content, and TN content), meteorological indicators (annual average temperature and precipitation), and management practices [straw incorporation and application rates of N, phosphorus (P), and potassium (K) fertilizers]. When meteorological data were unavailable in the literature, the missing information was obtained from the China Meteorological Data Network (https://data.cma.cn). The grouping of the influencing factors included in the meta-analysis is presented in **Table 1**. The criteria for factor classification were as follows: the annual mean temperature was divided into two categories using 13°C and 14°C as thresholds, while the annual average precipitation was classified based on 500 and 800 mm; soil classification was based on nutrient grading standards from the Second National Soil Survey and existing literature (Islam et al., 2022; Liu et al., 2022), combined with practical production conditions, to establish soil nutrient grades for this study.

**Table 1:** Subgroup classification of factors affecting crop yield.

Influencing factors	Index	Subgroup		
		1	2	3
Fertilization	Fertilization type	Chemical fertilization	Organic fertilization	Combined application
Soil indicators	Chemical indicators	TN, TP, SOM, AK, AP, AHN		
	Biological indicators	SMBC, SMBN, UA, SA, PA		
Climate factors	MAT/°C	≤13	13–14	>14
	MAP/mm	≤500	500–800	≥800
Soil base properties	pH	≤6	6–8	>8
	SOM/(g·kg^−1^)	≤10	10–15	>15
Trial duration	Years	0–30		
Fertilizer type	Organic–inorganic combined fertilizer rate			
N application rate (kg·ha^−1^)	Wheat	≤120	>120–225	>225
	Maize	≤150	>150–225	>225
P application rate (kg·ha^−1^)	Wheat	≤60	>60–120	>120
	Maize	≤45	>45–90	>90
K application rate (kg·ha^−1^)	Wheat	≤30	>30–60	>60
	Maize	≤30	>30–60	>60
Organic manure application rate (kg·ha^−1^)	Wheat	≤7,500	>7,500–15,000	>15,000
	Maize	≤6,000	>6,000–9,000	>18,000

TN, total nitrogen; TP, total phosphorus; SOM, soil organic matter; AK, available potassium; AP, available phosphorus; AHN, alkali-hydrolyzable nitrogen; SMBC, soil microbial carbon; SMBN, soil microbial nitrogen; UA, urease activity; SA, sucrase activity; PA, phosphatase activity.

### 2.2 Data calculation

As one of the key parameters in meta-analysis, when only SE is provided in the literature, SD is derived from SE using Equation 1, based on the relationship between SE, SD, and the number of replicates (*n*):

(1) $$SD=SE\sqrt{n}$$

If treatment group data are lacking, substitute with 1/10th of the corresponding experimental group or control group data (Luo et al., 2006). All data underwent standardization prior to analysis, with fertilizer application rates uniformly standardized. In field trials, fertilizer rates were adjusted based on application depth and soil bulk density. When application depth was unspecified, a default depth of 0–20 cm was used (Li, 2022). Soil organic carbon (SOC) data were converted to soil organic matter (SOM) using Equation 2 (Luo et al., 2006):

(2) SOM=SOC*1.724

The value 1.724 represents the conversion factor between SOM and SOC.

Logarithmic response ratios (RRs) were employed to evaluate the extent of the impact exerted by different fertilizer applications in the experimental treatments on soil nutrients and crop yields. For a given indicator in the study, the logarithmic response ratio (*R*) represents the ratio of the mean value (*X*_a_) in the treatment group to the mean value (*X*_b_) in the control group (Li, 2022), calculated using Equation 3:

(3) $$LnR=Ln\frac{Xa}{Xb}=LnXa-LnXb$$

The 95% confidence interval (CI) was calculated using 9,999 iterations of repeated sampling. For yield comparisons between no fertilization and various fertilization treatments, the effect was considered insignificant if the 95% CI included zero. If the minimum value exceeded zero, fertilization significantly increased the yield; conversely, if the maximum value was below zero, fertilization significantly decreased the yield (*P* < 0.05). To better characterize the magnitude of the fertilization effects on yield, the yield effect value ln*R* was converted to the relative change rate *M* using Equation 4 (Duan, 2022).

(4) M=(EXP(LnR)−1)*100%

### 2.3 Statistical analysis

In this study, data aggregation was performed using Microsoft Excel 2013 software. Normal distribution analysis was conducted with SPSS version 22.0. Meta-analysis was executed using the MetaWin 2.1 software. If *P >*0.05, indicating no heterogeneity in the data, the fixed-effects model was selected; if *P <*0.05, the random-effects model was used (Wallace et al., 2017). The contribution of factors affecting crop yield was assessed using the “Random Forest” package in R software (Liu et al., 2014). Graphical representations were generated using Origin 2021 software.

## 3 Results

### 3.1 Effects of different fertilization measures on wheat and maize yields

**Figure 3:**
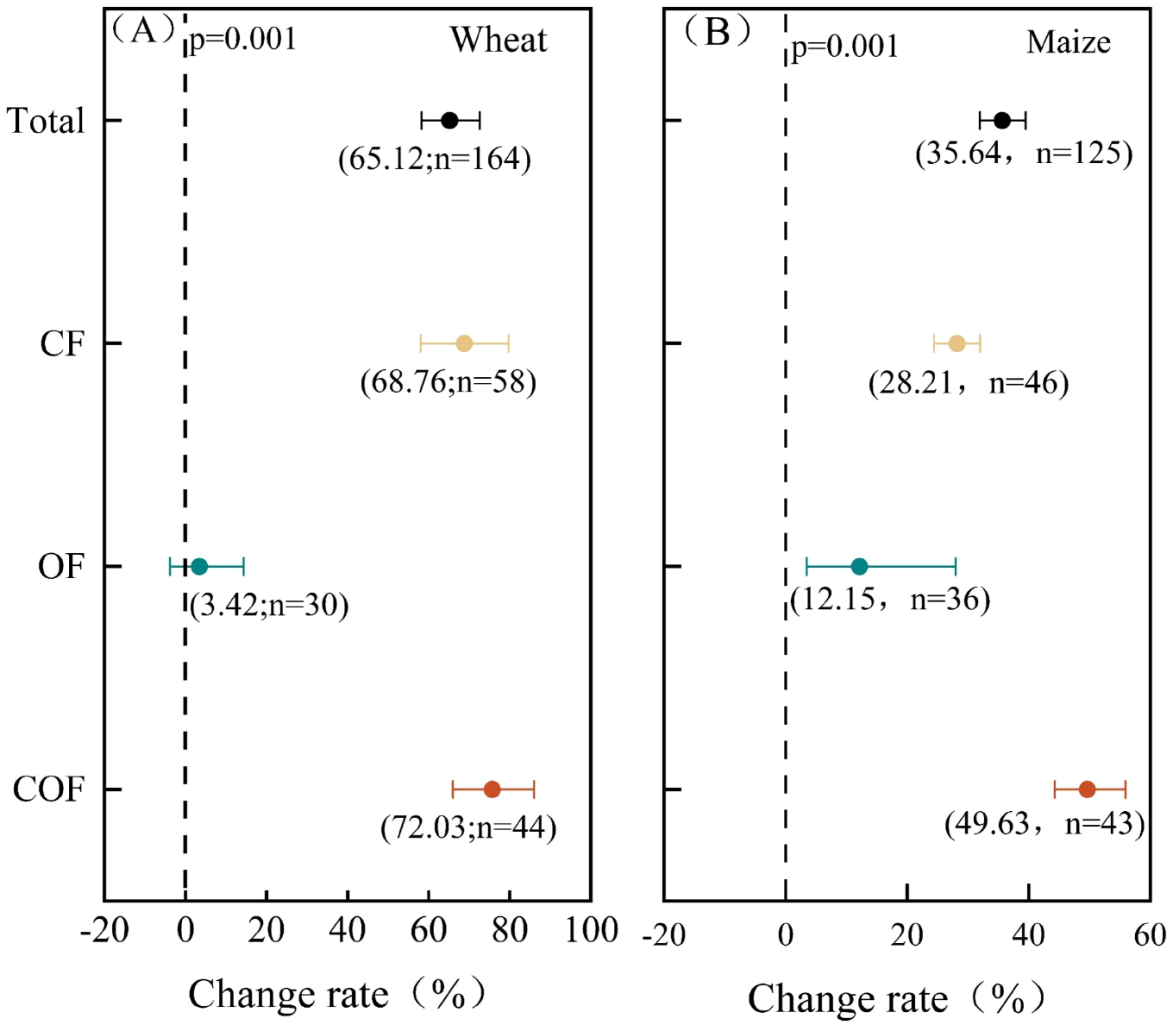
Crop yield responses to different types of fertilization. **(A)** Wheat. **(B)** Maize. The dots and error bars represent the mean percentage change and 95% CI, respectively. If the 95% CI does not overlap with the vertical 0 line (*P* < 0.05), the table is statistically significant (the same as the figure below).

The yield responses of wheat and maize on the North China Plain to different fertilization treatments are shown in **Figure 3**. Compared with the CK control (no fertilization), fertilization increased the average yields of wheat and maize by 65.12% and 35.64%, respectively. The combined application of organic and inorganic fertilizers resulted in the greatest yield increases for wheat and maize, at 72.03% and 49.63%, respectively. Chemical fertilizer alone produced the next highest increases, at 68.76% and 28.21%, respectively, whereas organic fertilizer alone yielded the lowest increases, at 3.42% and 12.15%, respectively. During the wheat season (**Figure 3A**), both chemical fertilizers alone and the organic–inorganic combination significantly increased wheat yield (*P* < 0.05) compared with the control and organic fertilizer alone. During the maize season (**Figure 3B**), although all three fertilization measures significantly increased maize yield (*P* < 0.05) compared with the control, the organic–inorganic combination significantly increased maize yield (*P* < 0.05) compared with the other treatments. However, there was no significant difference in yield increase between the application of chemical fertilizer alone and that of organic fertilizer alone. These results suggest that the combined application of chemical and organic–inorganic fertilizers has a stronger yield-enhancing effect on wheat than on maize and that the application of organic fertilizer alone is comparatively more effective during the maize season. This difference may be attributed to variations in environmental conditions and nutrient uptake characteristics during the growth stages of wheat and maize.

### 3.2 Effects of the fertilization measures on soil nutrient enhancement

**Figure 4:**
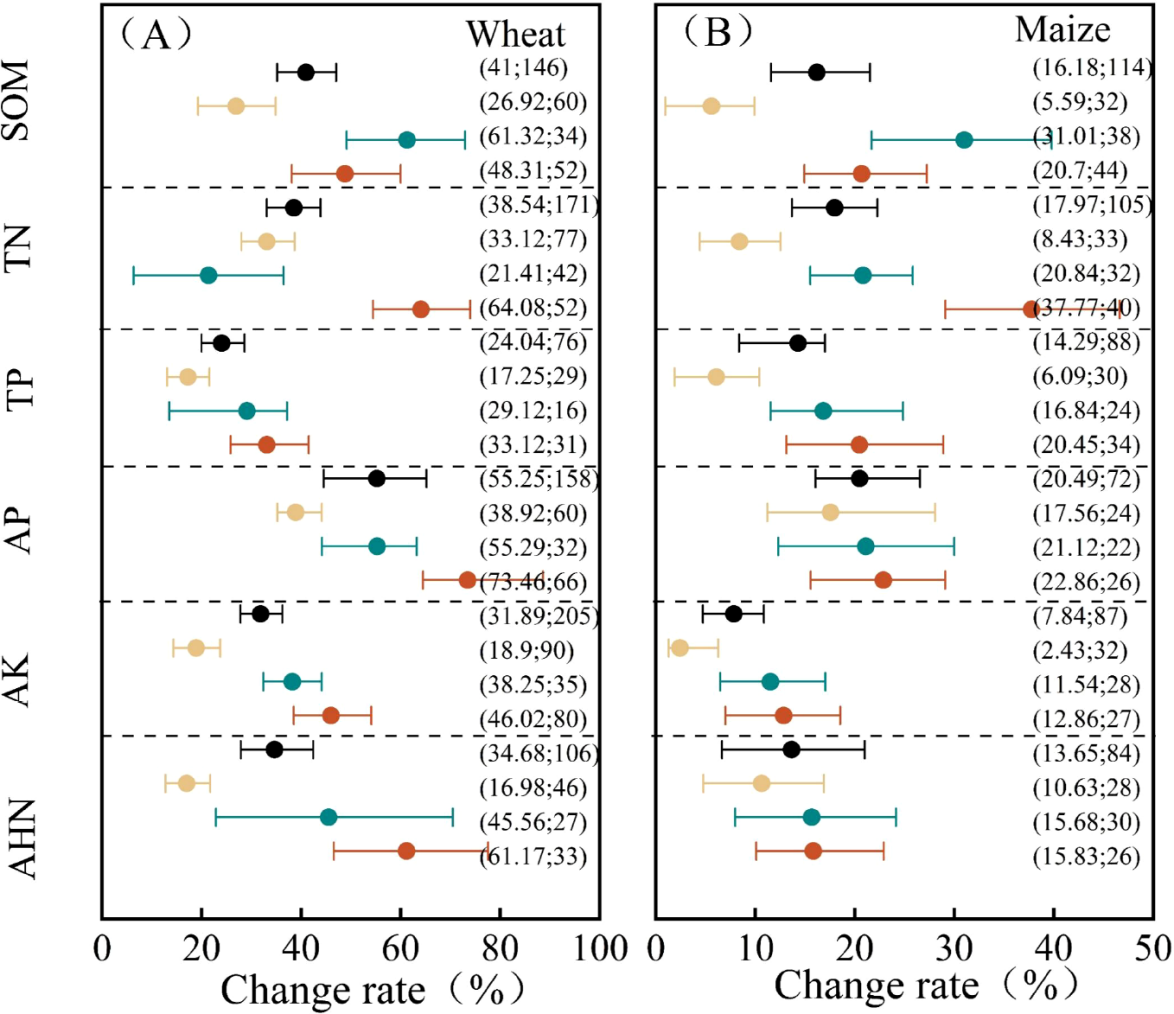
Soil nutrients’ responses to different fertilization practices at crop harvest. Black represents the total effect, yellow represents chemical fertilizer alone (CF), green represents organic fertilizer alone (OF), and red represents combined organic and inorganic fertilizer application (COF) (the same as the figure below). **(A)** represents wheat, and **(B)** represents maize.

During the wheat harvest period, all soil nutrients increased under the fertilization treatments (**Figure 4A**). Fertilization increased soil organic matter content by an average of 41%. Among the treatments, organic fertilizer resulted in the greatest increase in SOM content at 61.32%, which was not significantly different from the 48.81% increase observed with the combined organic–inorganic fertilizer application. Both of them were significantly higher than the 26.92% increase observed with chemical fertilizer alone. The average increase in TN content was 38.54%. The combined application of organic and inorganic fertilizers produced the highest increase in TN content (64.08%), which was significantly greater than the increases from either chemical or organic fertilizers alone. The average increases in soil TP, AP, AK, and AHN followed the order: combined application > organic fertilizer alone > chemical fertilizer alone. The increase in soil TP content under the combined organic–inorganic fertilizer application was 33.12%, significantly higher than the 17.25% increase observed with chemical fertilizer alone. The average increase in soil AP content was 55.25% with no significant differences among the treatments. Fertilization increased soil AK and AHN contents by 31.89% and 34.68%, respectively. For both, the combined application of organic and inorganic fertilizers and the application of organic fertilizers alone resulted in significantly higher increases than that of chemical fertilizers alone.

During the maize harvest period, the nutrient growth trends under different fertilization treatments were similar to those observed during the wheat season (**Figure 4B**), although the rates of increase were lower than those observed during the wheat season. Organic fertilizer alone resulted in the greatest increase in SOM content, at 31.01%, followed by the combined organic–inorganic fertilizer application at 20.70%. Both treatments significantly outperformed chemical fertilizer alone. The average increases in soil TN, TP, and AK contents due to fertilization were 17.97%, 14.29%, and 7.84%, respectively. Both the organic–inorganic mixed application and organic fertilizer alone produced significantly higher increases than chemical fertilizer alone. The increases in soil AP and AHN contents were 20.49% and 13.65%, respectively, with no significant differences among the fertilization treatments.

### 3.3 Effects of fertilization on soil microbial activity

**Figure 5:**
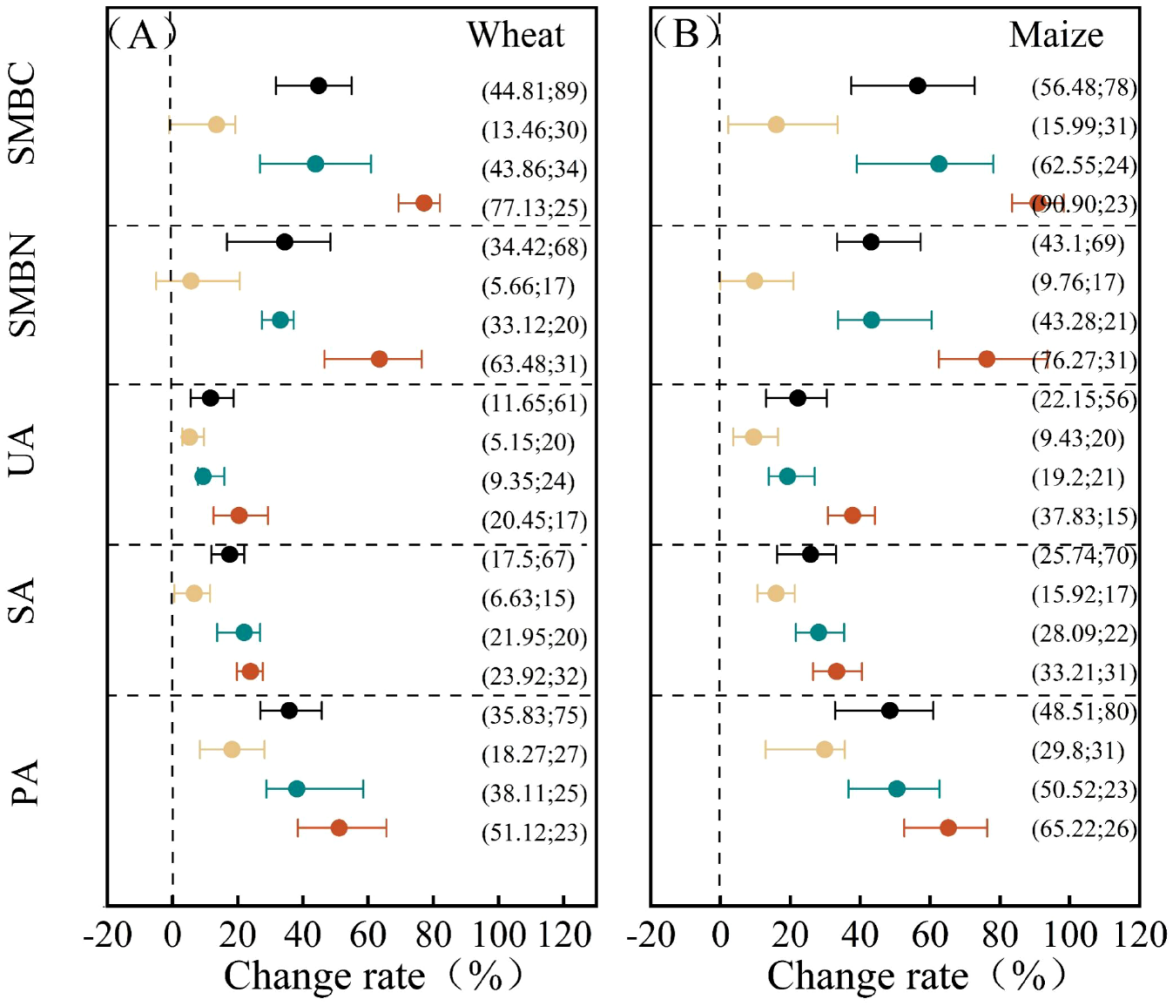
Responses of soil microorganisms and enzyme activities to different fertilization practices. **(A)** represents wheat, and **(B)** represents maize.

During wheat harvest, the order of increase for soil microbial biomass carbon (SMBC), soil microbial biomass nitrogen (SMBN), sucrase activity (SA), urease activity (UA), and phosphatase activity (PA) under different fertilization treatments was as follows: combined organic–inorganic fertilizer > organic fertilizer > chemical fertilizer (**Figure 5A**). All fertilization treatments enhanced both SMBC and SMBN, with average increases of 44.81% and 34.42%, respectively. The combined organic–inorganic fertilizer treatment produced the greatest increases, at 77.13% for SMBC and 63.48% for SMBN, followed by the organic fertilizer alone treatment, which increased SMBC and SMBN by 43.86% and 33.12%, respectively. The chemical fertilizer alone exhibited the weakest enhancement effect. The improvement rates for SMBC and SMBN under different fertilization indicated that SMBC increased more than SMBN. The combined application of organic and inorganic fertilizers significantly outperformed the single application of organic fertilizers, which, in turn, significantly exceeded the single application of chemical fertilizers. The average increase in soil UA content across the different fertilization treatments was 11.65%. The combined application of organic–inorganic fertilizer increased UA by 20.45%, organic fertilizer alone by 9.35%, and chemical fertilizer alone by 5.15%. The combined application of organic–inorganic fertilizer significantly exceeded the chemical fertilizer alone. Fertilization also increased soil SA and PA by 17.5% and 35.83%, respectively. The combined application of organic–inorganic fertilizer increased these values by 23.92% and 51.12%, whereas the application of organic fertilizer alone increased them by 21.95% and 38.11%, respectively. Both the application of organic–inorganic fertilizer and organic fertilizer alone were significantly more effective than the chemical fertilizer alone.

During the maize harvest period, the trends in microbial biomass and enzyme activity across the fertilization treatments mirrored those observed in wheat (**Figure 5B**), with the maize season exhibiting greater increases in SMBC, SMBN, and enzyme activity than the wheat season. The average increases in soil SMBC and SMBN across the different fertilization treatments were 56.48% and 43.10%, respectively. The application of combined organic–inorganic fertilizers resulted in the highest increases, at 90.90% for SMBC and 76.27% for SMBN, whereas organic fertilizer alone increased SMBC and SMBN by 62.55% and 43.28%, respectively. The increases observed with combined organic–inorganic fertilizer application were significantly greater than those with organic fertilizer alone, which, in turn, were significantly greater than those with chemical fertilizer alone. The order of increase in soil UA, SA, and PA was as follows: combined organic–inorganic fertilizer > organic fertilizer > chemical fertilizer. UA was significantly higher with organic–inorganic fertilizer application than with chemical fertilizer alone, whereas SA and PA were significantly higher with both organic–inorganic and organic fertilizer applications than with chemical fertilizer alone.

### 3.4 Effect of fertilization duration on the yield increase trend

**Figure 6:**
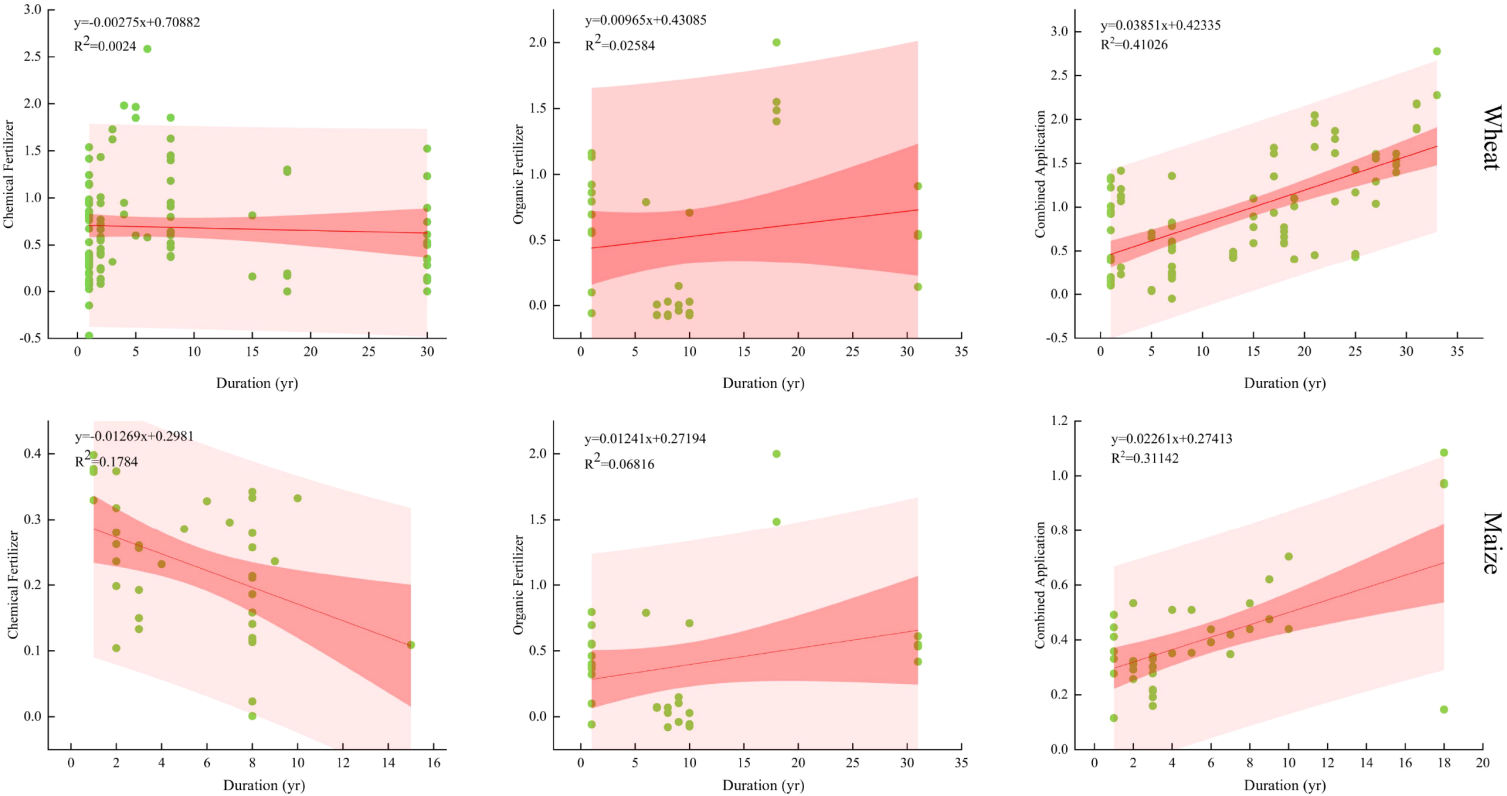
Crop yield responses to different fertilization measures over time. Note: The horizontal axis represents the number of years of continuous fertilization.

Under identical fertilization conditions, both crops exhibited consistent yield responses. **Figure 6** presents a scatter plot with fitted 95% confidence intervals. This equation represents a multiple linear regression model describing yield growth patterns across different fertilization cycles. When using chemical fertilizers alone, the yield increase trend gradually weakened with extended fertilization cycles, with maize showing a greater decline than wheat. Wheat yields are projected to cease increasing after 257.75 years, while maize yields stop increasing after 23.49 years. Conversely, when organic fertilizer was applied alone, the yield increase trend strengthened with extended fertilization cycles, with maize showing a greater increase than wheat. Both mixed application of organic and inorganic fertilizers and sole application of organic fertilizer exhibited upward trends, with the mixed fertilization showing superior yield enhancement effects compared to sole organic application.

### 3.5 Analysis of factors affecting fertilizer effects on crop yields

#### 3.5.1 Impact of meteorological factors

**Figure 7:**
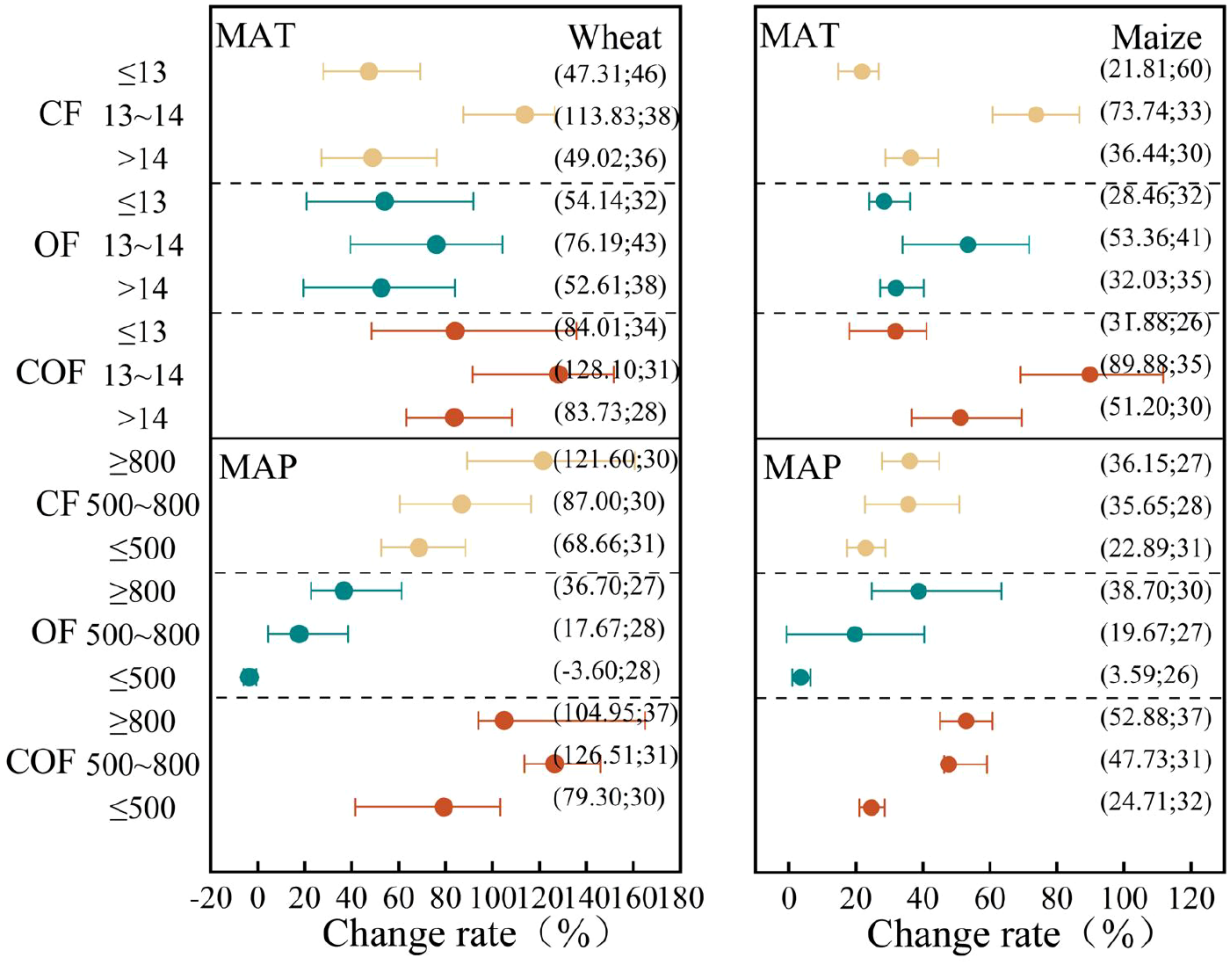
Crop yield response to annual temperature and precipitation under different fertilization practices.

The annual mean temperature in the North China Plain generally ranges between 10°C and 15°C (**Figure 7**). For wheat and maize, the greatest yield increases were observed where the temperature ranged from 13°C to 14°C, regardless of whether chemical fertilizer, organic fertilizer, or the combination was applied. Crops grown at 13°C–14°C produced significantly higher yields than those in other regions when chemical fertilizer was applied alone (*P* < 0.05). Similarly, maize yields at 13°C–14°C were significantly higher than those from regions with temperatures below 13°C (*P* < 0.05). Yield differences for other treatments were not significant across regions with different annual mean temperatures.

The application of chemical fertilizers and organic fertilizers alone resulted in yield increases with increasing annual mean rainfall. Under combined organic–inorganic fertilization, wheat yield increased most significantly by 126.51% where the annual mean rainfall ranged between 500 and 800 mm, significantly higher than at ≤500 mm. Maize yield peaked at 52.88% where the annual mean rainfall exceeded 800 mm, with yields at 500–800 mm also significantly higher than at ≤500 mm.

#### 3.5.2 Influence of soil base properties

**Figure 8:**
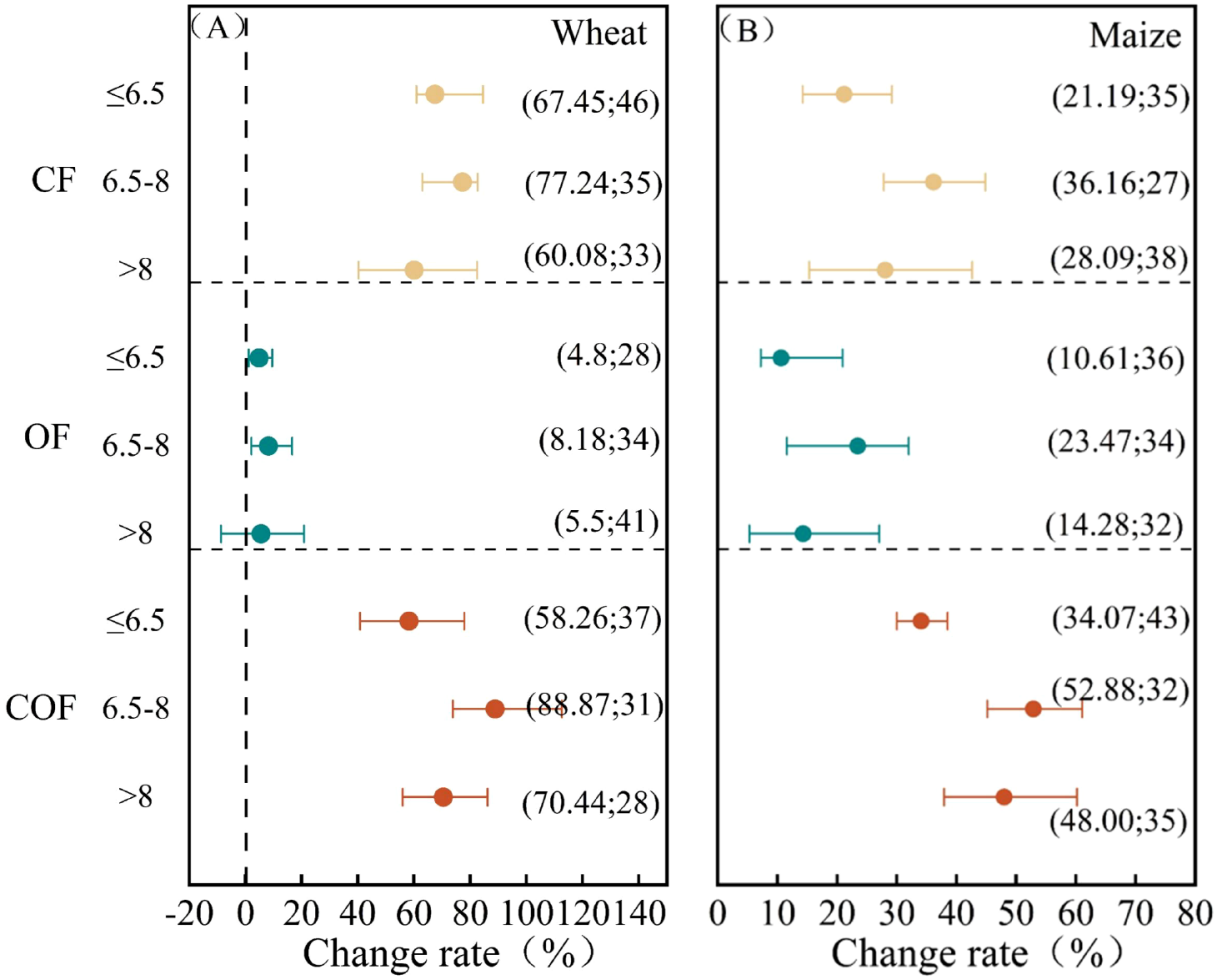
Crop yield responses to soil base pH under different fertilization practices. **(A)** represents wheat, and **(B)** represents maize.

Soil base pH affects wheat and maize yields (**Figure 8**). For both crops, all fertilization treatments resulted in the greatest yield increases where the pH ranged between 6 and 8.

**Figure 9:**
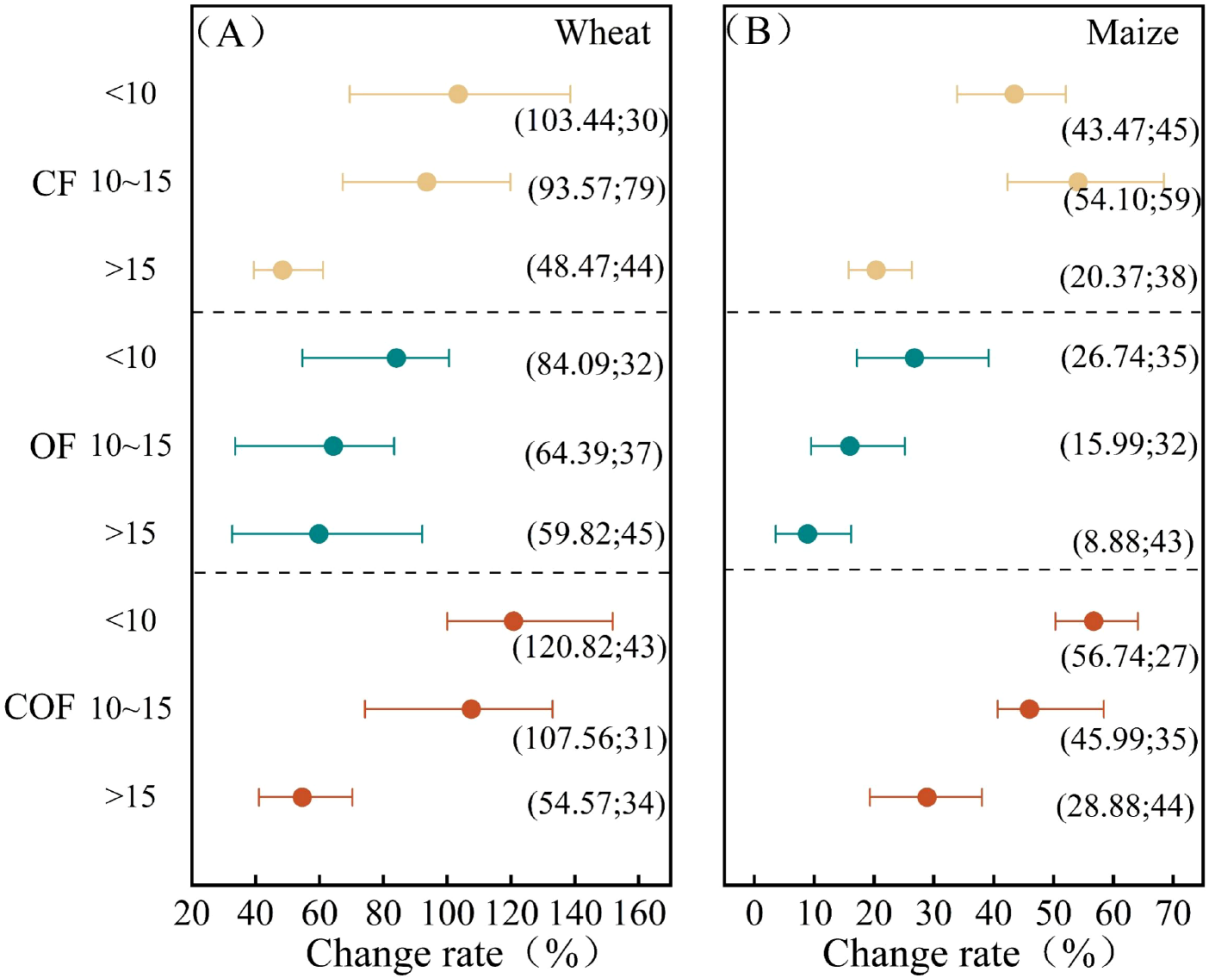
Crop yield responses to SOM under different fertilization practices. **(A)** represents wheat, and **(B)** represents maize.

The base SOM exerted similar effects on wheat and maize yields (**Figure 9**). For both wheat and maize, yield increases resulting from the application of chemical fertilizer alone, organic fertilizer alone, and combined organic–inorganic fertilizer application were similar within the same base SOM content range. The combined organic–inorganic and organic fertilizer alone produced the greatest yield increases, where organic matter content was below 10 g·kg^−1^, whereas chemical fertilizer alone resulted in the highest yield increase at 10–15 g·kg^−1^ organic matter content. The magnitude of this increase was greater for wheat than for maize. This means that fertilization helps to produce higher yields in fields with low SOM.

### 3.6 Analysis of contributing factors to fertilization effects on crop yields

**Figure 10:**
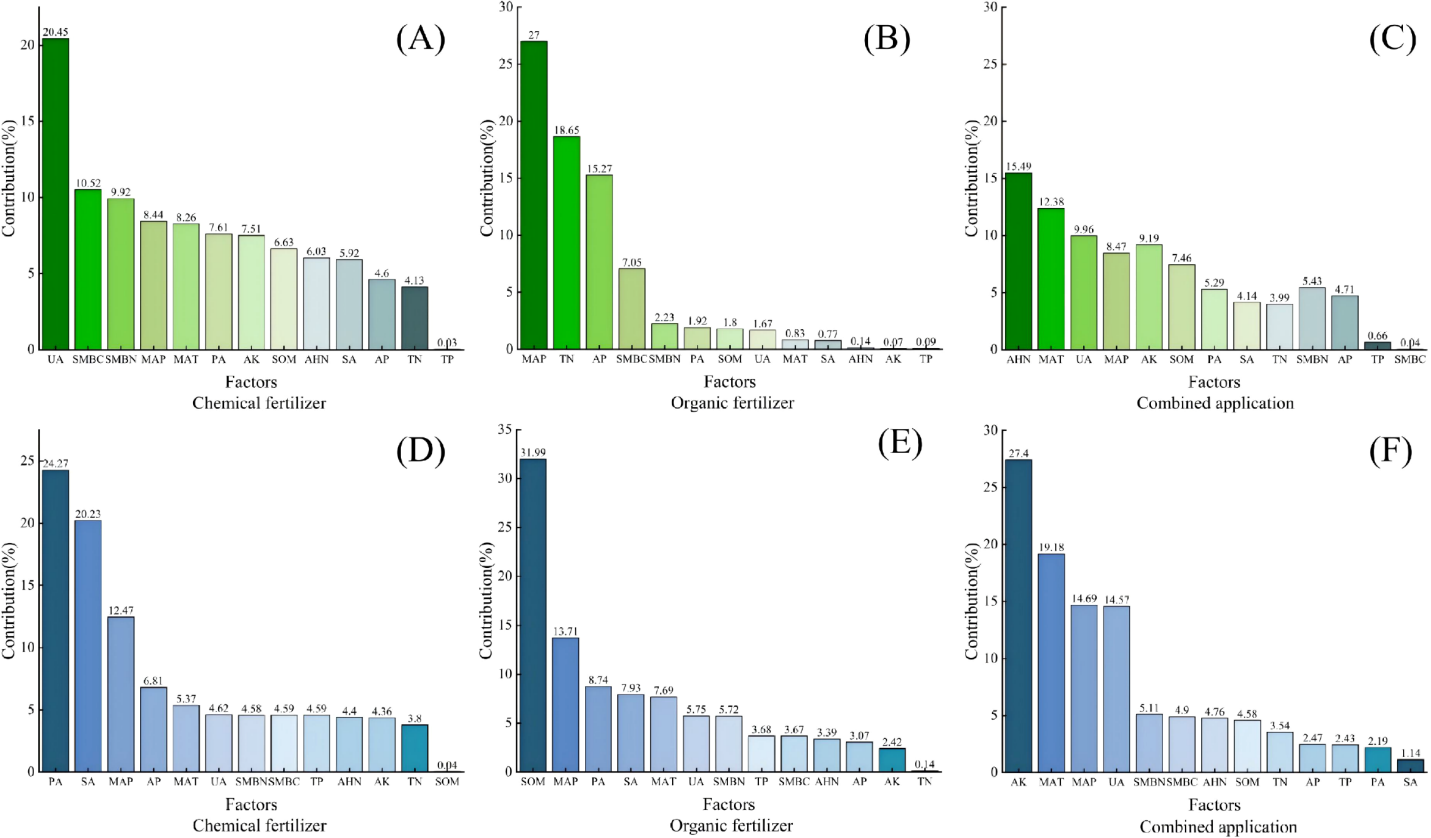
Contribution of factors to the effects of fertilizer on crop yields. **(A–C)** The contribution of each factor to wheat yield under chemical fertilizer, organic fertilizer, and combined organic–inorganic fertilizer applications. **(D–F)** The contribution of each factor to maize yield under chemical fertilizer, organic fertilizer, and combined organic–inorganic fertilizer applications.

When applying chemical fertilizers (**Figures 10A,D**), soil enzyme activity (UA, SA, PA) contributed 33.99% to wheat yield and 49.12% to maize yield, ranking first in terms of yield effect contribution. This suggests that fertilizers can directly enhance crop yields by accelerating nutrient transformation through the activation of soil enzymes. Furthermore, soil enzyme activity contributed 15.13% more to maize yield than to wheat yield, suggesting a more pronounced enzyme-promoting effect on maize than on wheat. This difference may be related to the higher nutrient requirements of maize and its greater dependence on enzyme-mediated nutrient transformations. Simultaneously, the contribution of microbial C and N to wheat (20.44%) was significantly higher than that to maize (9.17%), indicating that wheat is more sensitive to nutrient supply mediated by microbial C and N.

Under organic fertilizer application (**Figures 10B,E**), soil nutrients contributed 36.02% to wheat growth and 44.69% to maize growth, reflecting the enhancement of soil nutrient reserves by organic fertilizers. Meteorological factors accounted for 27.83% of the variation in wheat nutrition, which was significantly higher than that in the chemical fertilizer treatment. This may be because wheat fields enriched with organic fertilizer are more sensitive to water and temperature conditions, necessitating greater attention to field moisture and temperature management. Conversely, the contribution of meteorological factors to maize nutrition (21.4%) was lower than that of enzyme activity (22.42%), likely because the utilization of organic fertilizer by maize is less affected by weather and depends more on enzymatic conversion.

Under the combined application of organic–inorganic fertilizer (**Figures 10C,F**), the contribution gaps among soil nutrients, enzyme activity, and meteorological factors were reduced. The contributions of microbial C and N (wheat 5.47% and maize 5.11%) were lower than those of chemical fertilizers (wheat 20.44% and maize 9.17%) and organic fertilizers (wheat 9.28% and maize 9.39%). This suggests that the combined application can coordinate the effects of various factors, reduce the reliance on any single factor, and enhance the yield stability. It can also be inferred that under combined application, nutrients are more readily absorbed directly by crops, and microbial C and N accumulation is less affected by competition.

### 3.7 Effects of different fertilizer application rates under combined organic–inorganic fertilization on crop yield

As shown in **Table 2**, the combination of 120–225 kg·ha^−1^ of nitrogen fertilizer, 60–120 kg·ha^−1^ of phosphorus fertilizer, 30–60 kg·ha^−1^ of potassium fertilizer, and 7,500–15,000 kg·ha^−1^ of organic fertilizer during the wheat season yielded the greatest increase in grain yield. For maize, the combination of 150–225 kg·ha^−1^ of nitrogen fertilizer, 45–90 kg·ha^−1^ of phosphorus fertilizer, 30–60 kg·ha^−1^ of potassium fertilizer, and 6,000–9,000 kg·ha^−1^ of organic fertilizer yielded the greatest increase in yield. Wheat exhibited higher yield increases than maize across all fertilizer treatments. Specifically, wheat yields were 18.91%–30.49% higher with nitrogen fertilizer, 17.49%–25.7% higher with phosphorus fertilizer, 14.68%–22.21% higher with potassium fertilizer, and 20.17%–28.93% higher with organic fertilizer compared to maize. It is evident that nitrogen fertilizer exerts the most significant impact on yield, while organic fertilizer demonstrates a more stable effect on yield.

**Table 2:** Effects of different fertilizer application rates on crop yields under organic–inorganic fertilizer combination application.

Fertilizer type	N fertilizer		P fertilizer		K fertilizer		Organic fertilizer	
Crop type	Wheat	Maize	Wheat	Maize	Wheat	Maize	Wheat	Maize
Application rate (kg·ha^−1^)	≤120	≤150	≤60	≤45	≤30	≤30	≤7,500	≤6,000
Change rate (%)	76.3	45.81	69.42	43.72	64.13	41.92	73.24	44.31
95% CI	55.34; 103.12	42.30; 48.56	50.53; 90.69	40.57; 46.83	45.63; 85.19	40.38; 45.10	50.17; 95.38	42.24; 47.52
Sample size	25	24	31	26	18	24	22	27
Application rate (kg·ha^−1^)	120–225	150–225	60–120	45–90	30–60	30–60	7,500–15,000	6,000–9,000
Change rate (%)	81.42	57.34	83.12	56.8	78.9	55.6	86.72	57.41
95% CI	70.45; 95.26	55.19; 60.37	70.38; 93.42	54.59; 66.41	70.67; 84.29	53.51; 58.57	80.69; 95.39	55.29; 64.59
Sample size	24	26	23	23	27	28	30	25
Application rate (kg·ha^−1^)	>225	>225	>120	>90	>60	>60	>15,000	>18,000
Change rate (%)	71.32	52.41	69.12	51.63	64.50	49.82	72.11	51.94
95% CI	61.37; 84.29	54.69; 55.29	59.29; 78.31	46.27; 56.17	53.63; 72.59	46.4; 61.43	56.31; 83.72	40.81; 56.28
Sample size	24	16	23	25	20	24	26	24

## 4 Discussion

### 4.1 Analysis of fertilizer effects on soil productivity, nutrients, and microorganisms

Crop yield is the most direct indicator of plant development (Wallace et al., 2016). Fertilization effectively enhances soil fertility and crop productivity, thereby increasing yields within a certain range (Breiman, 2001). Meta-analyses indicate that improvements in soil fertility induced by fertilization significantly enhance crop yields (Ren et al., 2023). Comparisons among chemical fertilizer alone, organic fertilizer alone, and combined organic–inorganic fertilizer revealed that, for both crops, yield increases followed the pattern: combined organic–inorganic application > chemical fertilizer > organic fertilizer (Li et al., 2022) (**Figure 3**). This is primarily due to the synergistic effect of the combined application, which balances the rapid nutrient release from inorganic fertilizers with the slow-release supply from organic fertilizers (Jiang et al., 2024). This balance significantly increases chlorophyll content during various growth stages, thereby enhancing photosynthesis, promoting dry matter accumulation, and improving yield formation (Hu et al., 2025). Compared with chemical fertilizers, organic fertilizers exhibit lower absorption rates, slower release, and reduced uptake by crops, which consequently affects crop yield and mineral content (Adekiya et al., 2022). Organic fertilizer increased maize yields by 8.73% more than wheat yields, likely due to the higher seasonal temperatures accelerating the nutrient release rates in soil for the absorption of maize.

Crop yield increases are closely related to soil nutrients, SMBC, SMBN, and enzyme activity. Under different fertilization treatments, soil nutrients significantly contributed to yield (**Figure 10**). Following organic fertilizer application, the increase in soil organic matter for wheat and maize was significantly higher than in chemical fertilizer treatments, while no significant difference was observed compared to organic–inorganic compound fertilizer treatments. The increment order for TP, AP, AK, AHN, SMBC, SMBN, and enzyme activity was the highest for organic–inorganic compound fertilizer, followed by organic fertilizer (**Figures 4**, **5**) (Hosseini Bai et al., 2022; Li et al., 2015). Nutrient accumulation at harvest under all fertilization treatments was greater in the wheat season than in the maize season, whereas microbial biomass carbon and nitrogen, along with enzyme activity, were higher in the maize season than in the wheat season. Maize (C_4_) exhibits a 30%–40% higher photosynthetic rate than wheat, accumulating photosynthetic products more rapidly. Over 90% of its root biomass is concentrated in the 0–25-cm layer, enabling swift capture of nutrients in the tillage layer and rapid biomass growth during the growing season. Wheat (C_3_) has a moderate photosynthetic rate and a deep-rooted system, with root biomass in the 50–75-cm layer being 2.9 times that of maize, indicating greater carbon sequestration potential (Hirte et al., 2018; Wang et al., 2025). Twenty-nine years of field data indicate that under identical fertilization regimes in the North China Plain, wheat in a wheat–maize rotation system removes approximately 25%, 25%, and 30% less nitrogen, phosphorus, and potassium, respectively, than maize. System analysis confirms significantly lower nitrogen, phosphorus, and potassium uptake rates in wheat compared to maize, although wheat exhibits a higher harvest index (Khan et al., 2020). Under identical fertilization methods and application rates, wheat exhibits significantly higher nutrient accumulation than maize (Sun et al., 2024). Wheat’s deep root system, coupled with high C/N ratios and slow decomposition of straw residues, leads to substantial retention of readily available nutrients in the plow layer. However, its growth period coincides with colder temperatures, limiting soil microbial biomass and enzyme activities like urease and sucrase. Maize, conversely, grows during the hottest months of the year. Rising temperatures accelerate soil organic matter decomposition, promoting microbial proliferation and biomass accumulation, significantly increasing soil bulk microbial biomass carbon and nitrogen content (Ma et al., 2019). The synergistic effect of organic–inorganic compound fertilization enhances organic matter content while enriching key nutrients like carbon, nitrogen, phosphorus, and potassium. This creates optimal growth conditions for microorganisms, significantly increasing urease, sucrase, and alkaline phosphatase activity (Gao et al., 2023).

Long-term fertilization alters soil properties, thereby affecting yield enhancement (**Figure 6**). Prolonged use of chemical fertilizers further lowers soil pH through nitrification (Ma et al., 2019), which inhibits microbial activity and damages soil structure. Organic fertilizers, rich in organic matter and beneficial microorganisms, produce abundant organic acids through microbial metabolism. This activates slow-release nutrients in the soil, extends the supply cycle of available nutrients, provides ample carbon and nitrogen sources for soil microorganisms, significantly increases SMBC and SMBN content, and enriches biodiversity (Mamo et al., 2021; Wang et al., 2023). Research confirms significant positive correlations between crop yields and soil available nitrogen, alkaline phosphatase activity, urease activity, protease activity, microbial biomass, and nitrogen content (Yu et al., 2025). This study (**Table 2**) found that mixed application of organic and inorganic fertilizers as an efficient fertilization strategy enables both wheat and maize to achieve optimal yields with nitrogen fertilizer application rates within the ranges of 150–225 kg·ha^−1^, phosphorus fertilizer 60–90 kg·ha^−1^, potassium fertilizer 30–60 kg·ha^−1^, and organic fertilizer 7,500–9,000 kg·ha^−1^. This approach not only achieves yield increases with reduced chemical fertilizer application but also improves soil physicochemical properties and biological activity.

### 4.2 Influence of basic soil properties

Soil pH plays a crucial role in nutrient supply, maintaining soil microbial diversity and regulating soil enzyme activity. Changes in soil pH directly impact the efficiency of material cycling and energy conversion within soil–crop systems (Yang et al., 2022) (**Figure 8**). Increased precipitation often lowers soil pH, potentially correlating with higher yield gains observed in areas of the North China Plain receiving ≥500 mm annual precipitation (**Figure 7**). Within this region, soil pH exhibits a significant negative correlation with both annual mean temperature and precipitation (Zhao et al., 2024). Furthermore, soil pH strongly influences crop uptake of available nutrients and significantly impacts soil microbial activity (Liu et al., 2021). Under acidic soil conditions (pH < 6.5), phosphorus is immobilized by aluminum and iron, while calcium, magnesium, and molybdenum are adsorbed by colloids, rendering them unavailable for root uptake and leading to nutrient deficiencies and yield reductions (Carkner et al., 2023). Research indicates that applying chemical fertilizers in combination with 2.5 t·ha^−1^ of organic fertilizer can raise the pH of acidic soils and increase rice yields (Ghosh and Devi, 2019). Applying chemical fertilizers with 7.5 t·ha^−1^ of organic fertilizer can significantly lower the pH of alkaline soils and boost wheat yields (Wang et al., 2023). For most crops, neutral soils provide optimal growth conditions with high mineral nutrient uptake efficiency (Enesi et al., 2023). This study found that wheat and maize exhibited maximum yield increases within a pH range of 6.5 to 8, potentially related to the predominantly alkaline soils of the North China Plain. Crop varieties typically possess salt tolerance, while factors like fertilization and environmental conditions can alter soil pH during wheat growth, thereby influencing yield.

SOM plays a central role in soil fertility and physicochemical properties, directly influencing soil functions (**Figure 9**) (Ghosh et al., 2022). When baseline SOM content is below 10 g·kg^−1^, both organic fertilizers and organic–inorganic compound fertilizers yield the greatest yield increases. Conversely, when SOM content ranges between 10 and 15 g·kg^−1^, chemical fertilizers alone achieve the highest yield benefits. Applying organic fertilizers or organic–inorganic composite fertilizers increases yields by improving soil properties and enhancing fertility (Masoudi et al., 2023). Chemical fertilizers alone only show yield-enhancing effects at higher SOM levels. As SOM continues to increase, soil matrix fertility improves, and the yield-enhancing effect of fertilization shows a diminishing trend (Ma et al., 2023).

## 5 Conclusions

Through a meta-analysis of 30 years of fertilization studies, including single chemical fertilizer application, organic fertilizer application, and combined organic–inorganic fertilizer application, on the North China Plain fields, the following conclusions were drawn:

1) Short-term application of chemical fertilizers and combined organic–inorganic fertilizers in the North China Plain fields boosted wheat yields more than maize yields. Maize yields increased more significantly than wheat yields under organic fertilizer application.

2) Fertilization yielded the greatest increase in fields where the base soil pH was between 6 and 8, SOM was below 15 g·kg^-^¹, the annual mean temperature was 13°C–14°C, and the annual precipitation exceeded 500 mm.

3) Long-term application of chemical fertilizers alone had shown slowing yield increases, while combined organic–inorganic fertilization could sustainably boost yields and reduce the impact of individual factors on production. Integrated fertilizer application rates for wheat and maize were uniformly recommended as follows: Nitrogen fertilizer application rates ranged from 150 to 225 kg·ha^−1^, phosphorus fertilizer from 60 to 90 kg·ha^−1^, and potassium fertilizer from 30 to 60 kg·ha^−1^, supplemented with 7.5–9 tons·ha^−1^ of organic fertilizer. This approach reduced chemical fertilizer use while stabilizing yields and enhancing soil fertility.

## Data Availability

The data analyzed in this study is subject to the following licenses/restrictions: Details can be provided upon request, subject to constraints such as privacy laws. Requests to access these datasets should be directed to 2993245499@qq.com.
